# Self-management of musculoskeletal hand pain and hand problems in community-dwelling adults aged 50 years and older: results from a cross-sectional study in a UK population

**DOI:** 10.1186/s12891-016-1276-4

**Published:** 2016-10-06

**Authors:** Helen Myers, Krysia Dziedzic, Elaine Nicholls, Peter Croft

**Affiliations:** Arthritis Research UK Primary Care Centre, Institute for Primary Care and Health Sciences, Keele University, Keele, Staffordshire ST5 5BG UK

**Keywords:** Musculoskeletal hand pain, Self-management, Primary care

## Abstract

**Background:**

Musculoskeletal pain is common in adults, with the hand being frequently affected. Healthcare services have the potential to be of benefit to adults with hand pain and problems, through promotion and facilitation of self-management.

**Methods:**

This paper explores existing self-management in a UK population of community-dwelling adults aged 50 years and over using data from surveys and a nested clinical cohort study. Self-management of hand problems was considered in three ways: self-directed treatment approaches used, adaptation behaviours adopted and choice to consult with a healthcare professional.

**Results:**

The treatment approaches most commonly used were ‘exercise/movement’ (*n* = 151, 69 %) and ‘resting’ the hands (*n* = 139, 69 %). The use of adaptation behaviour was widespread: 217 (99 %) people reported using one or more adaptation behaviours. Under half of survey respondents who reported hand pain (*n* = 783, 43 %) had consulted a healthcare professional about their problem during the last year: the lowest rate of consultation was for occupational therapy (*n* = 60, 3 %).

**Conclusions:**

Self-directed treatment and adaptation behaviours were widespread in adults aged 50 years and over with hand problems, but consultation with a healthcare professional was low.

## Background

Hand pain and hand problems are common in community-dwelling adults aged 50 years and over [1], and with an increasing older population the absolute number of people with these problems is likely to rise. Maintaining hand function and preventing functional limitation in older adults is important for independence, quality of life and well-being [2, 3]. Reduced hand strength is a predictor of future disability [4], and has also been proposed as a marker for generalised frailty and reduced health-related quality of life [2]. Previous work has demonstrated that severe weakness is common in adults aged 50 years and over with hand problems, and increases with age [5]: this weakness translates into functional difficulties with activities such as opening new jars and picking up large objects [5]. The relationship between decreased hand strength in adults aged 65–79 years and loss of independence in activities of daily living has previously been noted [3]. Despite the severity of hand symptoms experienced, and the degree of functional limitation reported, little is known about how people with hand problems manage their symptoms and functional limitation in the community. A recent qualitative study [6] did however identify that people with hand osteoarthritis (OA) use a variety of strategies (for example, planning, compensation or circumvention) in order to keep actively performing valued activities. These strategies were mostly self-directed.

Self-management has been found to be strongly related to health-related outcomes in primary care consulters [7]. Clinical guidelines, largely based on clinician expert consensus recommend self-management techniques such as joint protection and hand exercises for people with hand OA [8]. Healthcare services have the potential to promote such self-management strategies and be of benefit to older people with hand problems and functional limitation. The importance of self-management is acknowledged in the NICE quality standard for osteoarthritis, which recommends referral to occupational therapy, assessment of impact on daily activities, and the promotion of self-management [9]. The quality standards recognise that self-management can improve patients’ experiences and health outcomes. Findings from a trial of self-management approaches for people with hand OA suggest that occupational therapists can support self-management in people with hand OA [10].

The decision to consult with a health problem may be driven by numerous complex factors, including perceptions of severity of the problem, interference with life, ability to cope and beliefs regarding treatment [11, 12]. The decision to consult is only one technique by which people self-manage their hand problems: a variety of other treatment approaches may also be used.

The aim of this paper is to identify the self-management techniques used by community-dwelling adults aged 50 years and over with a potential healthcare need for their musculoskeletal hand problem. Self-management will be considered in three ways: treatment approaches used (as described in international guidelines [8]), adaptation behaviours adopted, and choice to consult with a healthcare professional.

## Methods

**Fig. 1:**
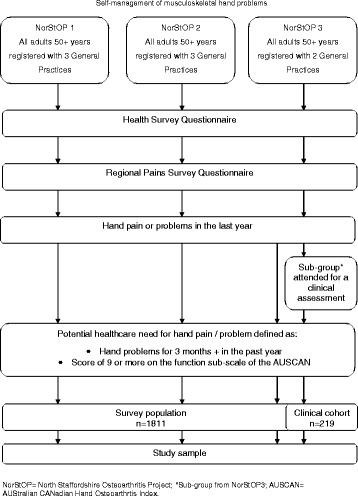
Simplified summary of recruitment procedures

Participants were 1811 people with a potential healthcare need for their hand pain or hand problems previously identified from a two-stage cross-sectional postal survey based within three linked longitudinal surveys (The North Staffordshire Osteoarthritis Project - NorStOP). The methods have been described in detail previously [13–15]. Briefly, a two stage survey was posted to all adults aged 50 years and over registered with eight general practices. Participants were eligible for this study if they completed a Health Survey questionnaire and a Regional Pains Survey questionnaire, indicated that they had experienced hand pain or hand problems in the previous 12 months, and met the definition of potential healthcare need (defined as persistent and limiting hand problems: hand problems lasting 3 months or more in the past year and a score of nine or more on the function sub-scale of the AUStralian CANadian Hand Osteoarthritis Index (AUSCAN) [16]). Information on self-management techniques were therefore self-reported. Recruitment procedures are summarised in Fig. 1.

Included in the 1811 eligible participants were 219 people who also underwent a detailed clinical interview and assessment as part of the Clinical Assessment Study of the Hand (CAS-HA), which was nested within the third NorStOP cohort [15]. Data from the 219 participants who attended for a clinical assessment were used to provide a detailed description of self-directed treatment approaches and adaptation behaviours. During the clinical interview, participants were asked which treatment approaches they were currently using (within the last month) for their hand pain, and, as a simple measure of outcome, whether they felt the treatment had worked well. They were also asked which adaptation behaviours they used. Participants were shown a laminated response card and were able to choose all behaviours which were applicable (for each behaviour, response options were ‘yes’ or ‘no’).

The treatment approaches question was adapted from a clinical interview schedule developed for musculoskeletal problems affecting the knee [17], and the options are shown in Table 1.

**Table 1:** Self-reported treatment approaches for hand pain and problems

Current^a^ use of:	
• Medication (over the counter, or prescribed)	
• Complementary therapies	
• Heat/warmth	
• Cold	
• Resting the hands	
• Exercise/movement	
• Massage	
• Creams/gels/rubs	
• Splints/supports	
• Positioning hands	

^a^within the past month

The adaptation behaviour question was developed from pilot work [18] and captured the following self-reported behaviours: use of gadgets, help from another person, avoidance (evading an activity), finding a different way of doing something, stopping/reducing (discontinuing or decreasing) activities, and taking longer.

The identification and description of consulters and non-consulters was undertaken in the whole cohort (*n* = 1811) via a self-report questionnaire. This asked participants whether they had consulted their GP, a physiotherapist, an occupational therapist or a hospital specialist within the last year ([15], adapted from [19]). Socio-demographic data (age, gender, socio-economic classification, living alone, and education) and hand affected were also obtained from this questionnaire.

### Function and pain measures

Functional ability was recorded in the whole cohort (*n* = 1811) by the function sub-scale of the AUStralian CANadian Hand Osteoarthitis Index (AUSCAN) [16] and the hand and finger function sub-scale of the Arthritis Impact Measurement Scale 2 (AIMS2) [20], with higher scores indicating more functional difficulty (0–36 and 0–10 respectively). Participants from the nested clinical cohort (*n* = 219) were additionally assessed for grip and pinch strength, simple objective function: Grip Ability Test [21] and crude functional hand movement – tested by observation of the ability to make a full fist [22] (all fingers tucked into the palm of the hand and the thumb fully flexed across the fingers, recorded as ‘yes/no’). The Grip Ability Test (GAT) consists of three timed sub-tests: 1. pulling a length of tubigrip onto the non-dominant arm, 2. putting a paperclip onto an envelope, and 3. pouring water from a jug. A maximum of 60 s is allowed for each subtest. The composite GAT score is calculated using the following formula: (time taken to complete sub-test 1 × 1.8) + time taken to complete sub-test 2 + (time taken to complete sub-test 3 × 1.8). A GAT score of >20 s is considered to indicate reduced hand function [21].

Pain was measured in the whole cohort (*n* = 1811) using self-report (AUSCAN pain sub-scale, [16], with higher scores indicating more pain (0–20).

### Analysis

Cross-sectional analysis was undertaken using data from the three combined baseline surveys and the nested clinical cohort. Key socio-demographic data were compared between the survey and clinical cohorts using summary statistics (frequencies and percentages for categorical data, and means and standard deviations for numerical data).

In the nested clinical cohort, the use of self-directed treatment and adaptation behaviour, stratified by age and gender were described using frequencies and percentages.

Odds ratios (with 95 % confidence intervals to provide an estimate of the statistical precision of our odds ratio estimates) were calculated using binary logistic regression to determine the effects of age and gender on treatment and adaptation behaviour. Results presented for gender are adjusted for age group and vice versa; the youngest age group (50–59 years) and male gender were used as the comparator groups. Analysis of covariance was used to explore whether differences in hand pain and function were independent of age and gender when comparing those consulting a health care professional to those who did not.

In the survey cohort, key demographic and severity characteristics (age, gender, AUSCAN hand pain, AUSCAN hand function, AIMS2 hand and finger function) were compared between those who did and did not consult a healthcare professional, using summary statistics (frequencies and percentages for categorical data, and means and standard deviations for numerical data). A logistic regression was carried out to assess whether there was a significant interaction between gender and age category on consultation rate (i.e. to establish whether any relationship between age category and consultation rate was different between males and females).

Pain and function were compared between consulters and non-consulters in the survey cohort, using means and standard deviations. A further comparison was carried out in the nested clinical cohort comparing grip and pinch strength, and observed hand function in consulters and non-consulters. Grip and pinch strength were compared to normative data [23, 24]. This comparison is reported as the number and percentage of participants who were below the normative values for each measure (only data for right hand presented). Statistical significance was determined using a chi-square test for categorical data and an independent samples *t*-test for numerical data. Analysis of covariance was used to determine the effect of age and gender on self-reported hand function and pain.

Within the nested clinical cohort, treatment approaches and adaptation behaviour were compared between consulters and non-consulters, using frequencies and percentages. Statistical significance was derived from the chi-square test.

Analysis was carried out using IBM SPSS Statistics for Windows, Version 21.0, Armonk, NY: IBM Corp.

## Results

Demographic data comparing the survey cohort (*n* = 1811) and the clinical cohort (*n* = 219) are presented in Table 2. Mean age and gender were similar between the cohorts. Differences were observed between the two groups: compared to the survey cohort, a larger percentage from the clinical cohort was employed in higher managerial/professional and lower managerial/professional occupations, attended full-time education and gained qualifications as an adult; a smaller percentage lived alone (Table 2).

**Table 2:** Socio-demographic data for the survey cohort (*n* = 1811) and the clinical cohort (*n* = 219)

	Survey cohort (*n* = 1811)	Clinical cohort (*n* = 219)
Age^a^	66.7 (9.6)	65 (8.2)
Gender (female)	1267 (70 %)	159 (73 %)
Socio-economic classification (1/2/3)^b^	257 (16 %)	46 (21 %)
	228 (15 %)	33 (15 %)
	1068 (69 %)	106 (48 %)
Living alone (yes)	496 (29 %)	40 (18 %)
Age left school^a^	14.9 (1.0)	15.2 (1.1)
Went on to full-time education after leaving school (yes)	177 (10 %)	33 (15 %)
Gained qualifications as an adult (yes)	501 (28 %)	90 (41 %)
AUSCAN function^a^	18.6 (6.3)	17.9 (6.0)
AIMS2 function^a^	4.0 (2.4)	4.0 (2.4)
AUSCAN pain^a^	10.0 (3.4)	9.9 (3.5)

^a^mean (standard deviation)

^b^the three class version of the National Statistics Socio-economic Classification (NS-SEC) was used [36]: 1 = higher managerial/professional and lower managerial/professional occupations; 2 = intermediate occupations; 3 = lower supervisory/technical, semi-routine and routine occupations; *AUSCAN* AUStralian CANadian Hand Osteoarthritis Index, *AIMS2* Arthritis Impact Measurement Scales 2; Scoring range for AUSCAN function = 0–36, with higher scores indication poorer function; Scoring range for AIMS2 hand and finger function = 0–10, with higher scores indicating poorer function; Scoring for AUSCAN pain = 0–20, with higher scores indicating more pain; data subject to missing data (although questionnaires were returned by 1811 people, not all of the questions within the questionnaires had been completed by every respondent)

### Self-directed treatment: Nested clinical cohort (*n* = 219)

The most common self-directed treatment approaches reported were ‘exercise/movement’ (*n* = 151, 69 %), ‘resting’ the hands (*n* = 139, 64 %), medication (*n* = 130, 59 %), ‘massage’ (*n* = 125, 57 %), ‘warmth/heat’ (*n* = 118, 54 %), and ‘positioning (*n* = 114, 52 %) (Table 3). A broadly similar percentage of males and females reported using these treatment approaches, with the exception of medication which was used by more females: f: *n* = 97 (61 %); m: *n* = 33 (55 %), and exercise, again used more by females: f: *n* = 117 (74 %); m: *n* = 34 (57 %). Differences were observed with age. The use of ‘resting’ the hands reduced with age, as did the use of ‘exercise/movement’. Overall, the use of ‘splints/supports’ decreased with age, although this was not a linear relationship, in that the use of ‘splints/supports’ increased slightly in the 60–69 year age group. Conversely, the use of ‘warmth/heat’ increased with age, although again this was not a linear relationship, in that the use of ‘warmth/heat’ decreased slightly in the 60–69 year age group (Table 3). After adjusting for gender, the only statistically significant age difference was observed for the use of splints, which were less likely to be used by those aged 70+ than those aged 50–59 (OR (95 % CI): 0.4 (0.1, 1.0) *p* = 0.05). After adjusting for age, the only statistically significant gender difference was observed for the self-reported use of exercise/movement: females had 2.2 times the odds of males for reporting use of exercise/movement (*p* < 0.0001) (Table 4).

**Table 3:** Observed frequencies of self-directed treatment and adaptation behaviour (clinical cohort)

	Clinical cohort (*n* = 219)				
	Age (years)			Gender	
	50–59 (*n* = 69)	60–69 (*n* = 83)	70+ (*n* = 67)	Males (*n* = 60)	Females (*n* = 159)
Self-directed treatment: *N* (%)					
Medication	43 (62 %)	45 (54 %)	42 (63 %)	33 (55 %)	97 (61 %)
Creams, gels, rubs	24 (35 %)	30 (36 %)	29 (43 %)	21 (35 %)	62 (39 %)
Splints/supports	15 (22 %)	23 (28 %)	6 (9 %)	9 (15 %)	35 (22 %)
Complementary	31 (45 %)	39 (47 %)	33 (49 %)	26 (43 %)	77 (48 %)
Warmth/heat	38 (55 %)	37 (45 %)	43 (64 %)	33 (55 %)	85 (54 %)
Cold	9 (13 %)	8 (10 %)	7 (10 %)	8 (13 %)	16 (10 %)
Resting	47 (68 %)	53 (64 %)	39 (58 %)	39 (65 %)	100 (63 %)
Exercise/movement	51 (74 %)	59 (71 %)	41 (61 %)	34 (57 %)	117 (74 %)
Massage	36 (52 %)	50 (60 %)	39 (58 %)	34 (57 %)	91 (57 %)
Positioning	38 (55 %)	40 (48 %)	36 (54 %)	30 (50 %)	84 (53 %)
Adaptation behaviour: *N* (%)					
Gadgets	42 (61 %)	48 (58 %)	51 (76 %)	33 (55 %)	108 (68 %)
Help	54 (78 %)	62 (75 %)	53 (79 %)	33 (55 %)	136 (86 %)
Avoidance	41 (59 %)	52 (63 %)	40 (60 %)	35 (58 %)	98 (62 %)
Different way	61 (88 %)	72 (87 %)	57 (85 %)	52 (87 %)	138 (87 %)
Stopping/reducing	37 (54 %)	50 (60 %)	42 (63 %)	35 (58 %)	94 (59 %)
Taking longer	56 (81 %)	66 (80 %)	55 (82 %)	47 (78 %)	130 (82 %)

Self-directed treatment and adaptation behaviour stratified by age and gender; self-directed treatment and adaptation behaviour are listed in the order in which they were asked; response options were ‘yes’ or ‘no’

**Table 4:** Age and gender effects on self-directed treatment and adaptation: odds ratios with 95 % confidence intervals

	Clinical cohort (*n* = 219)		
	Age (years)		Gender
	OR (95 % CI) for 60–69	OR (95 % CI) for 70+	OR (95 % CI) for females
Self-directed treatment:			
Medication	0.7 (0.4, 1.4)	1.0 (0.5, 2.0)	1.3 (0.7, 2.3)
Creams, gels, rubs	1.1 (0.5, 2.1)	1.4 (0.7, 2.9)	1.2 (0.6, 2.2)
Splints/supports	1.4 (0.7, 3.0)	0.4 (0.1, 1.0)*	1.7 (0.8, 3.9)
Complementary	1.1 (0.6, 2.1)	1.2 (0.6, 2.3)	1.2 (0.7, 2.2)
Warmth/heat	0.7 (0.3, 1.2)	1.5 (0.7, 2.9)	0.9 (0.5, 1.6)
Cold	0.7 (0.3, 1.9)	0.8 (0.3, 2.2)	0.7 (0.3, 1.8)
Resting	0.9 (0.4, 1.9)	0.6 (0.3, 1.2)	0.9 (0.5, 1.7)
Exercise/movement	0.9 (0.4, 1.9)	0.6 (0.3, 1.2)	2.2 (1.2, 4.1)*
Massage	1.4 (0.7, 2.7)	1.3 (0.6, 2.5)	1.0 (0.6, 1.9)
Positioning	0.8 (0.4, 1.4)	1.0 (0.5, 1.9)	1.1 (0.6, 1.9)
Adaptation behaviour:			
Gadgets	0.9 (0.5, 1.8)	2.1 (1.0, 4.4)	1.7 (0.9, 3.2)
Help	0.9 (0.4, 2.0)	1.1 (0.5, 2.5)	4.8 (2.4, 9.4)***
Avoidance	1.2 (0.6, 2.2)	1.0 (0.5, 2.0)	1.2 (0.6, 2.1)
Different way	0.9 (0.3, 2.3)	0.7 (0.3, 2.0)	1.0 (0.4, 2.4)
Stopping/reducing	1.3 (0.7, 2.5)	1.5 (0.7, 2.9)	1.0 (0.6, 1.9)
Taking longer	0.9 (0.4, 2.0)	1.1 (0.6, 2.6)	1.2 (0.6, 2.6)

*OR* odds ratio, *CI* Confidence interval; self-directed treatment and adaptation behaviour are listed in the order in which they were asked; response options were ‘yes’ or ‘no’

*p = 0.05; ***p ≤ 0.001

The majority of respondents felt that one or more of the treatment approaches they reported had worked well (*n* = 158, 72 %). This was similar between the genders. An increase in reporting that treatment had worked well was observed for the group aged 60–69 years, but this decreased in those aged 70+: (50–59, *n* = 48 (70 %); 60–69, *n* = 66 (80 %); 70+, *n* = 44 (66 %)). The use of medication was most frequently rated as working well by participants (*n* = 82, 37 %). Other commonly used treatments, such as ‘warmth/heat’, ‘exercise/movement’, ‘resting’ the hands, ‘massage’ and ‘positioning hands’, were felt to work well by approximately 25 % of participants, with no differences of note by gender or age.

### Adaptation behaviour: nested clinical cohort (*n* = 219)

The use of adaptation behaviour was widespread: 217 (99 %) participants used one or more behaviours. The most frequently reported behaviour was ‘finding a different way of doing something’, which was used by 190 (87 %) participants. The least frequently reported behaviours were ‘stopping/reducing activities’ (*n* = 129, 59 %) and ‘avoidance’ (*n* = 133, 61 %). There were no differences in gender or age, with the exception that females were more likely than males to report ‘using gadgets’ and ‘asking for help’, and those in the 70+ age group reported ‘using gadgets’ and ‘stopping/reducing activities’ more frequently than those in the 50–59 age group (Table 3). After adjusting for age, the only statistically significant gender difference was observed for ‘asking for help’: females had 4.8 times the odds of males for asking for help (*p* < 0.0001) (Table 4).

### Self-reported consultation: whole cohort (*n* = 1811)

Self-reported consultation for hand problems within the last year were as follows: 783 (43 %) any healthcare professional; 635 (35 %) GP (of which 363 (20 %) GP only); 290 (16 %) hospital specialist; 228 (13 %) physiotherapist; and 60 (3 %) occupational therapist. Of the subsample of 219 people who underwent a clinical interview and assessment, a similar pattern was observed: 96 (44 %) any healthcare professional; 80 (37 %) GP (of which 39 (18 %) GP only); 33 (15 %) hospital specialist; 32 (15 %) physiotherapist; and 11 (5 %) occupational therapist.

Demographic data for consulters and non-consulters were broadly similar and are presented in Table 5. The majority of consulters were right hand dominant (*n* = 682, 87 %), with 293 (38 %) reporting bilateral hand problems.

**Table 5:** Socio-demographic data for the survey population with hand problems, stratified by consultation (*n* = 1811)

	Not consulted HCP past year (*n* = 1028)	Consulted HCP past year (*n* = 783)
Mean (sd) age	67.7 (9.7)	65.4 (9.4)
Gender – female	736 (72 %)	531 (68 %)
Socio-economic classification (1/2/3)^a^	153 (17 %)/133 (15 %)/603 (68 %)	104 (16 %)/95 (14 %)/465 (70 %)
Living alone (yes)	281 (29 %)	215 (29 %)
Mean (sd) age left school	14.9 (1.0)	15.0 (1.0)
Went on to FT ed (yes)	109 (11 %)	68 (9 %)
Adult qualifications (yes)	294 (29 %)	207 (27 %)
Most problematic hand ^b^	445 (44 %)/208 (21 %)/348 (35 %)	304 (40 %)/164 (22 %)/293 (38 %)

*HCP* healthcare professional, *sd* standard deviation

^a^the three class version of the National Statistics Socio-economic Classification (NS-SEC) was used [36]: 1 = higher managerial/professional and lower managerial/professional occupations; 2 = intermediate occupations; 3 = lower supervisory/technical, semi-routine and routine occupations; *FT ed* full time education

^b^right/left/both; data subject to missing data (although questionnaires were returned by 1811 people, not all of the questions within the questionnaires had been completed by every respondent)

Overall, rates of self-reported consultation with a healthcare professional for hand problems within the last year decreased with age (50–59 *n* = 256, 50 %; 60–69 *n* = 271, 45 %; 70+ *n* = 256, 37 %), a difference which was statistically significant (*x* ^2^ = 21.3, df. 2, *p* < 0.001). This pattern was observed for both males and females, and there was no statistically significant difference between the genders (test for interaction *p* = 0.149) (data not shown).

Those who had consulted a healthcare professional had poorer objectively measured hand function (GAT and ability to make a full fist), and self-reported hand function (AUSCAN and AIMS2) than those who did not consult. Grip and pinch strength were marginally higher in consulters compared to non-consulters, but these differences were not statistically significant (Table 6). The majority of participants registered grip and pinch strength below normative values (*n* = 200, 91 % and *n* = 198, 88 % respectively for right hand). Compared to consulters, more non-consulters registered hand strength below normative values. These differences were only statistically significant (*p* < 0.05) for right pinch strength. Consulters from the population surveys had statistically significantly worse self-reported hand function (AUSCAN and AIMS2), and pain (AUSCAN) than non-consulters (all *p* < 0.001) (Table 6). These statistically significant findings remained after adjusting for age and gender (all *p* < 0.001) (data not shown).

**Table 6:** Comparison of pain and function in consulters and non-consulters (clinical cohort and survey population)

	Clinical cohort			
	All^1^ (*n* = 219)	Consulted HCP past year (*n* = 96)	Not consulted HCP past year (*n* = 123)	Statistical Significance
R Grip strength lbs^a^	33.4 (23.8)	35.6 (24.2)	34.8 (20.4)	*p* = 0.46^b^
Number (%) below average (normative data)	200 (91 %)	84 (88 %)	116 (94 %)	*p* = 0.07^c^
R Pinch strength lbs^a^	6.8 (6.7)	8.2 (4.0)	7.7 (4.0)	*p* = 0.34^b^
Number (%) below average (normative data)	192 (88 %)	79 (82 %)	113 (92 %)	*p* = 0.03^c^
GAT (secs)^a^	36.8 (18.1)	40.3 (21.5)	34.1 (14.5)	*p* = 0.02^b^
Unable to make a fist R	50 (23 %)	31 (32 %)	19 (15 %)	*p* = 0.01^c^
	Survey population			
	All^2^ (*n* = 1811)	Consulted HCP past year (*n* = 783)	Not consulted HCP past year (*n* = 1028)	Statistical Significance
AUSCAN function^a^	18.6 (6.3)	20.3 (6.5)	17.5 (5.9)	*p* < 0.001^b^
AIMS2 function^a^	4.0 (2.4)	4.6 (2.5)	3.7 (2.2)	*p* < 0.001^b^
AUSCAN pain^a^	10.0 (3.4)	11.2 (3.4)	9.3 (3.3)	*p* < 0.001^b^

*All* ^*1*^ 219 with a potential healthcare need from the nested clinical cohort, *All* ^*2*^ 1811 with a potential healthcare need from the survey cohort, *HCP* healthcare professional, *R* right, *GAT* Grip Ability Test, *AUSCAN* AUStralian CANadian Hand Osteoarthritis Index, *AIMS2* Arthritis Impact Measurement Scales 2

^a^mean (standard deviation)

^b^derived from independent samples *t*-test

^c^derived from Chi-square test; data for AUSCAN and AIMS2 subject to missing data (although questionnaires were returned by 1811 people, not all of the questions within the questionnaires had been completed by every respondent); Data for grip and pinch strength and GAT were checked for completeness in the clinic and are therefore subject to minimal missing data; A GAT score of > 20 s is considered to indicate reduced hand function [21]; Scoring range for AUSCAN function = 0–36, with higher scores indication poorer function; Scoring range for AIMS2 hand and finger function = 0–10, with higher scores indicating poorer function; Scoring for AUSCAN pain = 0–20, with higher scores indicating more pain

Within consulters (*n* = 783) there was no statistically significant difference observed in demographic characteristics and self-report measures between those who consulted an occupational therapist in the past 12 months and those who did not (Tables 5 and 6).

### Comparison between consulters and non-consulters in relation to self-directed treatment and adaptation

Without exception, the use of self-directed treatments and adaptation behaviours were reported more frequently in consulters than in non-consulters (Table 7). This was statistically significant for all treatments (range: *p* = 0.05 to *p* = 0.001) with the exception of 'complementary therapies' and 'warmth/heat'; and for all adaptation behaviours (range: *p* = 0.04 to *p* = 0.02) except the use of ‘gadgets’ and ‘asking for help’ (Table 7).

**Table 7:** Self-directed treatment and adaptation in those with hand problems, stratified by consultation (clinical cohort)

	Clinical cohort (*n* = 219)		
	Consulted HCP past year (*n* = 96)	Not consulted HCP past year (*n* = 123)	Statistical Significance^b^
Self-directed treatments			
Medication^a^	69 (72 %)	61 (50 %)	*p* = 0.001
Creams, gels, rubs	48 (50 %)	35 (29 %)	*p* = 0.001
Splints/supports	26 (27 %)	18 (15 %)	*p* = 0.023
Complementary	50 (52 %)	53 (43 %)	*p* = 0.186
Warmth/heat	58 (60 %)	60 (49 %)	*p* = 0.087
Resting	69 (72 %)	70 (57 %)	*p* = 0.022
Exercise/movement	73 (76 %)	78 (63 %)	*p* = 0.045
Massage	63 (66 %)	62 (50 %)	*p* = 0.024
Positioning	59 (62 %)	55 (45 %)	*p* = 0.038
Adaptation behaviour			
Use of gadgets	63 (66 %)	78 (63 %)	*p* = 0.735
Help	75 (78 %)	94 (76 %)	*p* = 0.766
Avoidance	66 (69 %)	67 (54 %)	*p* = 0.032
Different way	89 (93 %)	101 (82 %)	*p* = 0.022
Stopping/reducing	64 (67 %)	65 (53 %)	*p* = 0.039
Taking longer	84 (88 %)	93 (76 %)	*p* = 0.027

Self-directed treatment and adaptation behaviour stratified by consultation with a healthcare professional within the previous year; Self-directed treatment and adaptation behaviour are listed in the order in which they were asked

*HCP* healthcare professional

^a^pain killers, anti-inflammatory drugs, other tablets

^b^derived from chi-square test

## Discussion

This paper describes self-reported self-management techniques used by community-dwelling adults aged 50 years and over with hand problems. Participants from a nested clinical cohort reported a variety of ways in which they self-managed their hand pain and hand problems. We found that exercise was the most common treatment reported. This was particularly so for women. Techniques such as ‘resting’ the hands were also reported commonly, and whilst ‘resting’ may be considered a beneficial self-management technique if used in conjunction with activity, emphasis may be better placed on ‘pacing’ as part of a self-management strategy, rather than on ‘resting’ the hands per se.

The widespread use of self-management techniques suggests that non-pharmacological treatments were frequently utilised, reflecting the guidelines for hand osteoarthritis (OA) (for example [8]). We did not establish whether participants had been advised by a healthcare professional to use these techniques, but given the low level of consultation it is likely that at least some people using these techniques had obtained information from other sources.

The use of adaptation behaviour was widespread. Generally, women were more likely to report using adaptation behaviours than men, especially asking for help. Proactive adaptation behaviours (e.g. finding a different way of doing something) were used most frequently, suggesting that people are motivated to remain independent. A recent qualitative study [6] exploring the problems with everyday activities experienced by people with hand OA, observed that people used proactive adaptation behaviours (e.g. planning, compensation), but when these strategies became ineffective, felt forced to stop, avoid or find an alternative activity. Despite the widespread use of self-directed treatment and adaptation behaviour in our study, participants still reported moderate to high levels of functional limitation.

It has been suggested that people with chronic health problems, such as musculoskeletal disorders, devise their own strategies of care over time and use them when needed, for example, a recent systematic review [25] identified that people with OA delay conservative treatment and opt for self-management. Differences between the genders in engaging with self-management has been identified: for example, a recent study [26] identified that men’s engagement with self-management appears sub-optimal and could be enhanced by ensuring that self-management techniques are congruent with key aspects of masculine identity. A meta-analysis [27] concluded that males of all ages, ethnicities and nationalities were less likely to seek help than females.

In our study, fewer than half of those with hand problems reported consulting a healthcare professional within the last year: only 16 % of these consultations were with an allied health professional (13 % with a physiotherapist and 3 % with an occupational therapist). Those who consulted reported statistically significantly higher levels of pain and functional limitation than those who did not. Objectively measured and observed hand function was also worse in those who consulted than those who did not. We observed that consultation decreased significantly with age, yet, as our previous findings noted, many symptoms and functional difficulties increased with age [5].

Disability is an important determinant of seeking help [11], with primary care being the first point of contact with healthcare services for people with musculoskeletal hand problems such as OA [28]. Although the majority of those who do consult are assessed and managed in primary care, it has been estimated that the vast majority of people with such problems either do not consult on a regular basis, or fail to consult their GP at all [29].

In the UK, consultation with a general practitioner is of particular importance as the GP is the gate-keeper to other healthcare services, such as occupational therapy. Those who do consult their GP for their hand problems may not be referred to allied health professionals. The reasons for this are likely to be varied, but may include GP knowledge about treatment offered by, the availability of, and access to, such services.

Barriers to seeking help may prevent people from consulting. OA is still considered by some clinicians (and some patients) to be an inevitable consequence of ageing, and as such, its impact on people’s lives may be trivialised [30]. Amongst clinicians, negative attitudes to OA and its management remain, with the unwarranted view persisting that ‘nothing can be done’ [31]. Despite the availability of a wide variety of treatments [8], medication is often the only treatment option recommended by GPs [32]. However, for long-term management, it is the treatment strategy least preferred by patients [33]. From a patient’s perspective, resignation to pain and disability as part of the normal ageing process [34], or previous unsatisfactory experiences, may dissuade consultation.

People may not consult on a regular basis because they have learned to cope with their problems using simple adaptations and over-the-counter analgesia; the former, it has been suggested, being used in favour of the latter [32]. The findings from this study partly support this observation, with the use of adaptation behaviour and medication being widespread in both consulters and non-consulters. Despite the widespread use of medication, only 37 % of participants felt that it worked well, and only a quarter of those using other treatment techniques reported that these worked well. Adaptation behaviour was not a substitute for consultation, in that adaptation was found in consulters as well as in non-consulters.

Congruent with a report which concluded that Europe’s men need their own health strategy [35], encouraging men to engage with self-management may be a potential priority target for intervention. Our findings suggest that consultation with allied health professions in the last year was low. Improving access to such services in primary care may be important, and the role and profile of these services needs to be promoted and developed in this setting to enhance the management of hand problems and reduce or prevent resultant functional limitation.

Future work needs to establish how potential healthcare need in community-dwelling adults aged 50 and over with hand problems might be most effectively met, particularly given the low-level of consultation. Knowledge of the natural course of hand problems over time will provide further information regarding the likely need for healthcare services. Such services need to be timely, appropriate and cost-effective and driven by the needs of the population. Given the current GP-driven referral system in primary care, thought needs to be given to how to make such services more accessible and how GPs can be educated about the scope of allied health professions.

### Limitations

The cross-sectional nature of our analysis means that temporal relationships cannot be established, for example, although the use of self-directed treatment and adaptation behaviour was seen more in consulters, it is not possible to speculate whether these self-management strategies were used prior to, or post-consultation. We observed that participants reported moderate to high levels of functional limitations despite widespread use of self-directed treatment and adaptation behaviour. This finding may be interpreted in one of two ways: these techniques are not effective in reducing pain or improving function, or, people only start to use self-directed treatment once their function and/or symptoms had worsened. The cross-sectional nature of this analysis therefore does not permit judgements to be made regarding the effectiveness of these techniques in maintaining hand function, or their role in preventing limitation occurring. Similarly, we do not know whether the level of functional limitations would have been higher if participants had not used these self-management strategies.

Recall of consultations over the duration of a year may have been imprecise, and respondents may not have correctly recalled which healthcare professional they had consulted, particularly in the case of therapists. This may have resulted in some over- or under-estimation of consultations.

The medication question did not differentiate between prescribed and over-the-counter medication: what the authors have ascribed to self-directed treatment may actually be a reflection of prescribed medication following a consultation. The question about what ‘worked well’ in relation to treatment approaches, was a crude measure lacking precise definition, and as such, was open to subjective interpretation by participants: some responding to this question may have viewed working well as ‘not making them worse’, whilst others may have interpreted it as ‘making them better’.

Self-directed management approaches were only collected in a relatively small sample. Despite those who attended for a clinical assessment having similar socio-demographic characteristics to those from the larger population who did not, those who attended for a clinical assessment may have been more willing to try self-directed management approaches than those who did not.

This analysis was exploratory in nature, with multiple statistical tests being carried out. The statistically significant results therefore need to be interpreted with caution. These results may reflect the availability and accessibility of services in North Staffordshire and therefore any generalisations should be considered carefully.

## Conclusions

The use of self-directed treatment approaches and adaptation behaviour in adults aged 50 years and over were widespread. Fewer than half of those identified as having a potential healthcare need for their hand problem reported consulting a healthcare professional within the last year. Although consulters tended to have worse function and more pain than non-consulters, there remained a large number of people who experienced pain and functional limitation who did not consult. Despite NICE recommendations [9], allied health professions saw only the tip of the iceberg of those with a potential healthcare need for their hand problems. Self-management is a key quality standard of care for OA [9], and existing strategies used by patients could be supported by allied health professions in a primary care setting. Future work will use longitudinal data to further investigate the temporal relationship between self-management approaches and hand function.

## Abbreviations

AIMS2: Arthritis Impact Measurement Scale 2
AUSCAN: AUStralian CANadian Hand Osteoarthritis Index
CAS-HA: Clinical Assessment Study of the Hand
CI: Confidence interval
df: Degrees of freedom
GAT: Grip Ability Test
GP: General practitioner
HCP: Healthcare professional
NICE: The National Institute for Health and Care Excellence
NorStOP: North Staffordshire Osteoarthritis Project
NS-SEC: National Statistics Socio-economic Classification
OA: Osteoarthritis
OR: Odds ratio
OT: Occupational therapist
SD: Standard deviation
SPSS: Statistical package for the social sciences
UK: United Kingdom
*x*^2^: Chi square
